# Controlled Pilot Intervention Study on the Effects of an AI‐Based Application to Support Incontinence‐Associated Dermatitis and Pressure Injury Assessment, Nursing Care and Documentation: Study Protocol

**DOI:** 10.1002/nur.22469

**Published:** 2025-04-16

**Authors:** Hannah Pinnekamp, Vanessa Rentschler, Khalid Majjouti, Alexander Brehmer, Michaela Tapp‐Herrenbrück, Michael Aleithe, Jens Kleesiek, Bernadette Hosters, Uli Fischer

**Affiliations:** ^1^ Department of Clinical Nursing Research and Quality Management, Nursing Department Hospital of the Ludwig‐Maximilians‐University (LMU) Munich Munich Germany; ^2^ Department Nursing Development and Nursing Research University Hospital of Essen Essen Germany; ^3^ Institute for Artificial Intelligence in Medicine (IKIM) Essen Germany; ^4^ sciendis GmbH Leipzig Germany; ^5^ Catholic University of Applied Sciences Munich (KSH) Munich Germany

**Keywords:** Artificial Intelligence, incontinence‐associated dermatitis, pressure injury, wound assessment, wound care

## Abstract

Artificial Intelligence (AI)‐based applications have significant potential to differentiate between pressure injuries (PI) and incontinence‐associated dermatitis (IAD), common challenges in nursing practice. Within the KIADEKU overall project, we are developing an AI‐based application to aid in the nursing care of PI and IAD and to facilitate personalized, evidence‐based nursing interventions. The KIADEKU clinical sub‐study described in this study protocol is a controlled, non‐randomized clinical pilot intervention study investigating the effects of the AI‐based application, fully developed in the KIADEKU overall project, on the duration of wound assessment, dressing change and documentation, guideline adherence, and nurse task load. The study utilizes a pre‐post design with two data collection periods. During the initial phase, we will observe and survey nurses in the control group as they provide conventional wound care without AI support to adult patients with PI or IAD in the pelvic area across eight wards at the LMU University Hospital. In the following intervention phase, the AI‐based application will assist nurses in wound assessment and deliver guideline‐based nursing interventions for documented wound types. Observations and surveys will be repeated. Measurements will include the duration of wound assessment, dressing changes, and documentation, adherence to wound care guidelines, and the accuracy of AI predictions in clinical settings, validated by an on‐site expert assessment. The survey will assess nurses’ task load and other covariates, such as professional experience, overall workload during the shift, and wound severity. Linear regression models will be used to analyze the effects of AI usage on the aforementioned aspects, taking into account these covariates. The accuracy of AI predictions regarding wound type and classification will be measured using the on‐site expert's assessment as the ground truth. The usability of the AI‐based application and standard clinical documentation systems will be evaluated further. The deployment of the AI application in clinical settings aims to reduce the duration of wound assessments, dressing changes, and documentation; decrease nurse task load; enhance guideline adherence in wound care; and promote AI utilization in nursing. German Clinical Trials Register (DRKS) (DRKS00031355). Registered on April 5th, 2023.

**Trial registration:**

German Clinical Trials Register (DRKS) DRKS00031355. Registered on April 5^th^ 2023.

**Patient or Public Contribution:**

Patient representatives contributed to the development of the AI‐based application through the use of Delphi methodology, as part of the KIADEKU qualitative sub‐study.

**Abbreviations**
AE: Adverse Event
AI: Artificial Intelligence
BMBF: German Federal Ministry of Education and Research
DRKS: German Clinical Trials Register
EPUAP: European Pressure Ulcer Advisory Panel
GLOBIAD: Ghent Global IAD Categorization Tool
IAD: Incontinence‐associated dermatitis
IKIM: Institute for Artificial Intelligence in Medicine
KI: Artificial Intelligence (German abbreviation)
KSH: Katholische Stiftungshochschule Munich University of Applied Sciences
LMU: Ludwig‐Maximilians‐University Munich
MIT: Department of Medical Technology and IT at LMU University Hospital
PI: Pressure Injury
SAE: Serious Adverse Event

## 1 Background

Artificial Intelligence (AI)‐based applications are increasingly used in clinical wound management, and show substantial potential for enhancing wound assessment, care, and treatment. They offer benefits such as standardized wound images, accurate wound measurements, identification of wound complications, support for classification and treatment decisions, and efficiencies in documentation (Dabas et al. 2023; Lau et al. 2022; Mohammed et al. 2022). However, most research primarily focuses on physicians as the end‐users of these applications, often overlooking the nursing perspective. These AI‐based applications are powered by AI models trained on large datasets to autonomously identify patterns and make predictions or decisions. The models use various algorithms and network architectures, frequently combining multiple models to improve performance.

Pressure injuries (PI) are a globally recognized clinical issue and a key indicator of quality in nursing care (European Pressure Ulcer Advisory Panel, National Pressure Injury Advisory Panel, & Pan Pacific Pressure Injury Alliance 2019; Gemeinsamer Bundesausschuss 2023). Without timely and effective treatment, PIs can lead to severe patient and economic burdens, including extended hospital stays and increased healthcare costs (Labeau et al. 2021).

Incontinence‐associated dermatitis (IAD) results from prolonged exposure to urine or feces, leading to skin inflammation (Gray et al. 2007). It is a prevalent condition among patients with incontinence in acute care, with studies reporting a prevalence of 42% to 45.7% (Campbell et al. 2016; Gray and Giuliano 2018). Clinically, IAD manifests as erythema, potentially accompanied by skin loss, and can include skin infections such as candidiasis (Gray et al. 2007). Pressure injuries, on the other hand, can range from non‐blanchable erythema with intact skin to complete skin destruction, extending through subcutaneous fat, muscle, and even bone (European Pressure Ulcer Advisory Panel, National Pressure Injury Advisory Panel, & Pan Pacific Pressure Injury Alliance 2019).

The visual similarities between IAD and PI, particularly in their early stages and common gluteal location, frequently lead to uncertainty and misclassification in nursing practice (Beeckman et al. 2008; LeBlanc et al. 2016). Despite the availability of validated assessment tools like the European Pressure Ulcer Advisory Panel (EPUAP) guidelines (European Pressure Ulcer Advisory Panel, National Pressure Injury Advisory Panel, & Pan Pacific Pressure Injury Alliance (2019)) and the Ghent Global IAD Categorization Tool (GLOBIAD), differentiating between these wound types remains a challenge for nurses (Beeckman et al. 2017, 2018; Francis 2019). Furthermore, studies suggest that nurses often possess limited knowledge about IAD (Barakat‐Johnson et al. 2022; Zhang et al. 2021), and misconceptions in assessing these wounds can lead to inappropriate intervention choices and delays (Beeckman et al. 2010). Beyond these assessment difficulties, challenges in wound documentation also persist. Research indicates that wound documentation frequently fails to capture all details of the assessment and care (Do et al. 2021; Gillespie et al. 2014; Li and Korniewicz 2013; Topaz et al. 2016). Issues such as irregular wound shapes, localization, and identifying exact wound margins can complicate measurement accuracy (Haghpanah et al. 2006; Keast et al. 2004). The use of low‐tech tools like paper rulers and depth probes often results in poor interobserver reliability and measurement inaccuracies (Haghpanah et al. 2006; Keast et al. 2004; Wang et al. 2017). Additionally, wound documentation is time‐consuming, particularly when photographing wounds. Digital cameras are employed for this purpose in clinical nursing practice at LMU University Hospital, and uploading these images to electronic patient records is often tedious and can be delayed by up to 2 days or more.

In other nursing areas, AI‐based technologies are already being used to support evidence‐based nursing practices (Ronquillo et al. 2021). Common challenges include a lack of technical and informatics skills (Abuzaid et al. 2022; Gerich et al. 2022) as well as ethical concerns (Robert 2019). To overcome these challenges and ensure AI applications are optimally adapted to the needs of professional nurses, it is crucial to involve nurses in the research and development processes (Gerich et al. 2022; Zhou et al. 2021). This involvement can greatly enhance the utility of AI for nurses and lead to improved patient care outcomes.

**Figure 1:**
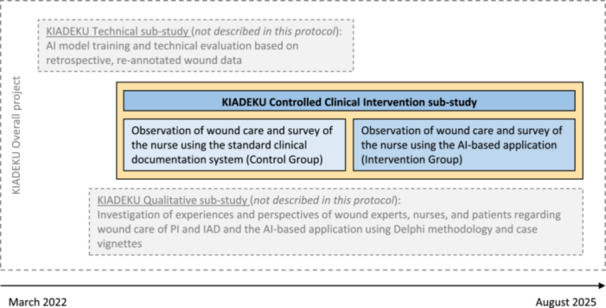
KIADEKU Overall project and sub‐studies.

In the KIADEKU (KI^1^‐IAD‐DEKUbitus^2^) research and development overall project (German Clinical Trials Register number (DRKS00029961)), we are developing an AI‐based application for digital image analysis of PI and IAD. This development is based on nursing‐relevant questions and is being conducted in cooperation with the Institute for Artificial Intelligence in Medicine (IKIM). As illustrated in Figure 1, the overall project encompasses a technical sub‐study, a qualitative sub‐study, and the controlled clinical intervention sub‐study described in this study protocol.

To develop the AI model in the technical sub‐study, a literature‐based minimal data set for wound documentation of PI and IAD was defined. Based on this, 1,555 existing wound images and associated characteristics were extracted from the hospital information systems of two German university hospitals. The study team validated, segmented, and annotated these images and characteristics, achieving consensus on wound type and classification. Subsequently, four AI network architectures were evaluated, and the model with the best performance for differentiating between PI and IAD was selected. This transformer‐based model's performance was systematically enhanced through additional methods of data pre‐ and post‐processing, augmentation techniques, and training methods. The performance of the developed AI model for binary differentiation of PI and IAD and wound classification was evaluated using accuracy and F1 score (Harmonic Mean of Precision and Recall) on a test data set. These processes are described in more detail in the technical sub‐study conducted by the Institute for Artificial Intelligence in Medicine (IKIM).

After the AI model training was completed, we integrated it into the WUNDERA® wound documentation application of the project partner sciendis GmbH and it is now being used in the clinical intervention sub‐study to assist nurses in differentiating, assessing, and documenting PI and IAD wounds, and to facilitate personalized, evidence‐based nursing interventions. In a further qualitative sub‐study, the project partners at University Hospital Essen involve patients, wound experts, and nurses from both hospitals in the development of the AI, exploring their experiences and perspectives on the AI‐based application using group Delphi methodology and case vignettes.

The objective of this controlled clinical intervention sub‐study is to assess the quantitative effects of the AI‐based digital wound management application, which was fully developed in the technical sub‐study, on nurses’ wound assessment, care, and documentation. This evaluation focuses on the duration of wound assessment, dressing changes and documentation, the task load of nurses, and guideline adherence compared to conventional care in a non‐randomized pre‐post intervention study. Additionally, we will determine the accuracy of the AI‐generated predictions of wound type and wound classification in the clinical setting, as well as the usability of the AI‐based application.

## 2 Method and Design

### 2.1 Objectives

The primary objective of this sub‐study is to explore the relationship between the use of the AI‐based application and several factors: the duration of wound assessment, dressing changes, and documentation; the nurse's task load; and adherence to guidelines. We will adjust for potential covariates such as wound severity, the nurse's professional experience, and the general workload of the shift. A secondary objective is to evaluate the accuracy of the AI predictions regarding wound type and classification in a clinical setting, as well as the usability of the AI‐based application and standard clinical documentation systems.

Our hypothesis, which we intend to test, is that the use of the AI‐based application will enhance the differentiation, assessment, and documentation of PI and IAD by reducing the time required for wound assessment, dressing changes, and documentation; lowering nurses’ task load; and improving guideline adherence.

### 2.2 Ethics Approval and Consent to Participate

The study has received approval from the Ethics Committee of the LMU Medical Faculty (process number 22‐0970), the Data Protection Officer of LMU Hospital (process number 1952) and the Staff Council of LMU Hospital. All procedures will be conducted in accordance with the Declaration of Helsinki, and any significant changes to the study protocol will require re‐approval from both the Ethics Committee and the Data Protection Officer. We will ensure compliance with all Ethics Committee requirements and obtain informed consent from all participants before enrollment. Should adjustments to the study protocol become necessary during the study, the study registration will be updated accordingly.

### 2.3 Design

The study is designed as a monocentric, non‐randomized, controlled pilot intervention study with a pre‐intervention data collection period followed by a data collection period during the deployment of the AI‐based application (intervention) on the wards. Due to the nature of the intervention, it is not possible to blind either participants or observers.

**Figure 2:**
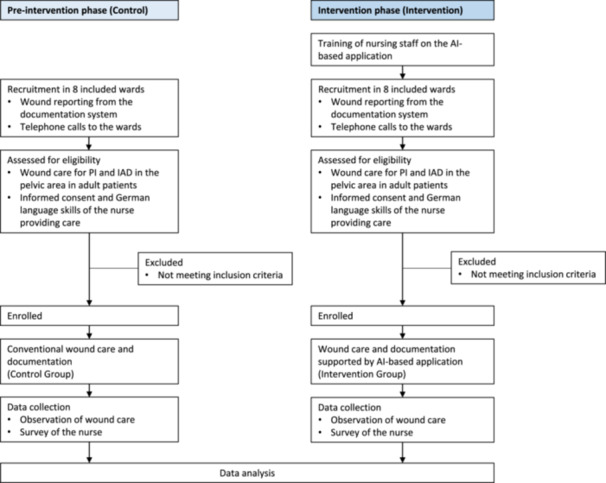
KIADEKU controlled clinical intervention sub‐study flow diagram.

In the pre‐intervention phase, we observe the wound care and documentation for PI and IAD according to hospital standards in the control group, using a standardized observation form, and survey the nurse using a standardized questionnaire (Figure 2). In the subsequent intervention phase, we train the nurses in the intervention group to use the AI‐based application, then observe and survey the wound care and documentation supported by the AI‐based application, consistent with the procedures used in the pre‐intervention phase (Figure 2). The reporting of the study protocol is based on the SPIRIT AI extension (Cruz Rivera et al. 2020).

### 2.4 Setting

We are conducting the study at LMU University Hospital, a center renowned for its high‐end medicine, nursing, medical innovation, and research. With over 2,000 beds, LMU Hospital is a highly advanced hospital that encompasses all fields of medicine.

For this study, we selected four intensive care units, one intermediate care unit, and three general wards. These wards were chosen due to their notably high incidence of patients with pressure injuries (PI) or incontinence‐associated dermatitis (IAD), reflecting the specific diseases treated and the demographics of their patient populations. These wards differ in terms of medical specialty and the number of staff, ranging from 33 to 54 employees (average 45.8) in the intensive care and intermediate care units, and from 15 to 28 employees (average 23) in the general wards. The nursing and ward managers agreed to participate in this study. In addition, consent was obtained from the Staff Council, the Data Protection Officer, and the Ethics Committee of the LMU Medical Faculty. Additionally, before the commencement of the study, we confirmed that the selected wards had adequate Wi‐Fi coverage to support the use of the AI‐based application on tablets and smartphones.

### 2.5 Participants and Eligibility Criteria

The study sample comprises adult volunteer nurses providing PI and IAD care for newly occurring wounds or pre‐existing wounds from newly admitted patients on the participating wards. Since IAD occurs solely in the pelvic area and the AI specifically aids in differentiating between the two wound types, only wounds located in the pelvic area are considered. All participating nurses must be fluent in German and are required to sign informed consent documents as part of the ethical clearance process. Exclusions apply to wound care for other types of wounds, patients under the age of eighteen, wounds outside the pelvic area, and patients who, according to the nurse's assessment, are in the preterminal or terminal phase. We also exclude intensive care patients who are too unstable for positioning for wound care or for whom life‐sustaining measures are being terminated. Additionally, nurses who do not give informed consent, do not speak fluent German, or are non‐registered nurses, trainees, and interns are excluded.

The inclusion and exclusion criteria for data used in AI training are detailed in the technical sub‐study conducted by the Institute for Artificial Intelligence in Medicine (IKIM). The input data for AI prediction includes the wound image captured in the application and additional patient data (such as immobility, incontinence, and sensibility assessments) entered by the nurse. Images are reviewed for suitability for prediction (e.g., exposure, image clarity), and, if necessary, users are requested to capture a new wound image.

### 2.6 Sample Size

Based on current methodologies, we performed an a priori sample size calculation using power analysis (Döring and Bortz 2016) with the G*Power 3 software (Faul et al. 2007). For this calculation, we assumed moderate effect sizes between dependent and independent variables (Cohen 1988), an alpha level of 0.05, and a power of 0.80. To test correlations between dependent variables (task load, time, guideline adherence) and four independent variables (use of the demonstrator, general load of the shift, professional experience, severity of wound) using multivariate models (multiple linear regression) under these assumptions, we will require a total of 85 nurses.

With a calculated sample size of 85 nurses and considering up to four independent variables, we anticipate obtaining meaningful results within a manageable timeframe and with the available resources. Accounting for a projected 10% dropout and missing rate, based on experiences from previous studies, we plan to recruit 95 nurses to ensure a sufficiently large sample for subsequent evaluation.

### 2.7 Recruitment

A dedicated electronic wound reporting system will be utilized to identify wounds that meet the inclusion and exclusion criteria. Due to the absence of an electronic reporting system for IAD and the likelihood that many wounds are not electronically documented, the research team will contact the included wards daily by telephone. They will inquire about new cases of PI or IAD or the admission of patients with existing PI or IAD wounds. Each patient is only included once in the study.

If wounds that meet the inclusion and exclusion criteria are identified, the study team will arrange for the next scheduled wound care appointment. At this appointment, the study team will inform the patient's nurse about the study. If the nurse in charge declines participation, no observation or survey will be conducted at that time. Still, the patient may be re‐enrolled at the next wound care appointment if the subsequent nurse agrees to participate. If the nurse consents in writing to participate, the study team will proceed with the observation of wound care and documentation. After completing the wound documentation, the nurse will complete an online questionnaire on a tablet, which includes questions about demographic data, general workload of the shift, task load, and the usability of the documentation system used. The study primarily targets new wounds and newly admitted patients with wounds. Due to organizational factors, there may be delays in some cases, including weekend admissions, nurses refusing to participate, and delayed documentation of new wounds.

To encourage participation, we engaged local wound experts and ward managers in the development of the digital system in the KIADEKU qualitative sub‐study and as key users during its implementation on the wards to enhance user acceptance. Additionally, nurses who agree to participate will receive vouchers as compensation for the extra effort involved in the study. These measures are anticipated to help achieve the sample size determined by the a priori sample calculation.

After careful consideration, obtaining patient consent was deemed unnecessary. This decision was based on the fact that wound management in the study aligns with standard practice, with the only variation being the documentation tool used. Furthermore, the study does not collect data that could identify patients. Nonetheless, the study team informs patients about the study before administering wound care.

### 2.8 Standard Care

In the pre‐intervention phase, we will observe the conventional wound care and documentation practices for PI and IAD in the selected wards. These practices are governed by internal hospital standard operating procedures for wound care and documentation, developed in accordance with the German national expert standards “Pressure Ulcer Prevention in Nursing” and “Nursing Care for Patients with Chronic Wounds” (Büscher et al. 2021; Schiemann 2009). These guidelines are available to nurses through a digital platform within the hospital. For electronic documentation of wounds, nurses capture wound images using a digital camera and upload these images to the computer‐based clinical documentation system, where they also enter additional details about the wound characteristics. No changes to the hospital infrastructure or processes related to wound care and documentation are anticipated during the study period.

### 2.9 Intervention

In the intervention phase, nurses will use the AI‐based application developed in the KIADEKU technical sub‐study (German Clinical Trials Register number DRKS00029961) on tablets and smartphones to provide wound care and documentation. The final version of the AI model is still under development and will be specified upon the publication of results. The AI‐based application used is a prototype demonstrator and has not been approved as a medical device. Therefore, this study is not a clinical evaluation under the Medical Device Regulation but a pilot and feasibility study to explore the potential of AI in nursing and support further development.

Before the intervention, nurses in the intervention group will receive training on how AI works, the relationship between the data they collect and the AI, and how to use the AI‐based application effectively. They will be trained to interpret the prediction probabilities displayed by the AI and manage incorrect predictions. It is emphasized that the AI‐based application is a prototype, not a fully developed product, and that AI predictions may be incorrect and should not replace expert nursing judgment at this stage.

During wound care, the nurse will use a tablet or smartphone to take a picture of the wound and enter additional patient information, such as incontinence status, sensibility, and mobility. The AI checks the image's suitability for prediction based on exposure and definition. If the image quality is deemed inadequate, the system prompts the user to retake the image. The application does not request any personal patient information that would allow identification, such as name, date of birth, or case number. The AI‐based application automatically measures the wound using a patch as a reference point, and predicts the wound type, classification, and documentation characteristics based on the wound image and additional patient data. Additionally, the system displays the AI‐calculated probability for each characteristic. The nurse reviews and, if necessary, revises the AI's predictions to ensure accurate wound documentation and appropriate subsequent interventions. Optionally, the application suggests nursing interventions from the hospital's existing wound care standard operating procedures relevant to the documented wound type and characteristics. In additional information fields, the AI‐based application provides detailed descriptions and definitions of wound types (e.g., EPUAP and GLOBIAD classifications) and further information on in‐house wound management standards (e.g., contraindications or user instructions for specific wound dressings).

The primary difference between the standard documentation system and the AI‐based application is that photography and documentation are conducted on‐site using tablets and smartphones, unlike in standard care where documentation occurs post‐care using fixed PCs and digital cameras. Additionally, in the AI‐based application, the wound is automatically measured, and the wound type and characteristics are pre‐populated by the AI model based on the wound image, which then must be validated and revised by the nurse if necessary. In contrast, the standard systems only offer the option of using the last known values. Furthermore, the AI‐based application provides additional information such as wound severity categories and appropriate nursing interventions for the documented wound, features that are absent in the standard system.

By automatically measuring the wound and pre‐populating wound characteristics, the AI is expected to decrease the time required for wound documentation. Nevertheless, the decision‐making process regarding the documented wound characteristics and subsequent wound management remains with the nurse, who must critically review, confirm, or edit the characteristics as necessary. The use of the AI‐based application is not expected to lead to performance errors or adverse events, as all predictions are verified and, if necessary, corrected by the nurse.

### 2.10 Measures

The measures of this study include the duration of wound assessment, dressing change, and documentation; the task load of the nurse during wound care and documentation; guideline adherence; and the accuracy of the AI predictions of wound type and classification in the clinical setting.

The duration of wound assessment, dressing change, and documentation is monitored during observation using standardized start and endpoints. The start point is defined as the moment dressing removal begins. If no dressing is present, the start point is the initiation of the first hands‐on wound care activity after patient positioning, such as a finger pressure test, measurement, or wound disinfection. Preparations, including patient positioning and material preparation, or interruptions, such as retrieving supplies from outside the room or responding to calls from other patients, are not included in the measurement. Documentation duration is measured separately since it does not always occur immediately after treatment. The start point is either opening the digital wound documentation or connecting the camera to the computer, and the endpoint is when the wound documentation is saved. It is also recorded whether a wound photograph is taken and whether the last known values are transferred from the existing documentation.

Guideline adherence is assessed using a set of five criteria identified during an expert workshop as relevant for evaluating guideline adherence in the context of wound care for PI and IAD. These criteria are derived from the “Prevention and Treatment of Pressure Ulcers” guideline published by the European Pressure Ulcer Advisory Panel (European Pressure Ulcer Advisory Panel, National Pressure Injury Advisory Panel, & Pan Pacific Pressure Injury Alliance 2019).

The selected criteria include performing a finger pressure test or the transparent disk method in the presence of redness (recommendation 9.1), accurately assessing wound type and classification (recommendation 9.2), applying wound edge protection in cases of macerated wound edges or surrounding skin (recommendation 3.1), and ensuring pressure relief for pressure injuries (recommendations 5.1 and 5.5). The response options for these criteria are binary: ‘No’ or ‘Yes or not required’. Sum scores are calculated from the responses, assigning a score of 1 to ‘Yes or not required’ and a score of 0 to ‘No’. Interrater reliability is also measured in the study. Additionally, the nurse's task load during wound assessment, dressing change, and documentation is evaluated using the standardized and validated NASA TLX questionnaire (Human Performance Research Group n.d.).

Other covariates measured include the nurse's professional experience, wound severity, and the general workload of the shift, which is assessed on a 5‐point scale relative to the average.

To assess the accuracy of the AI predictions of wound type and wound classification in the clinical setting, the wound is independently assessed on‐site by a member of the study team, separate from the nurse's assessment and the AI prediction. This assessment serves as the ground truth for calculating the accuracy of the AI predictions regarding wound type and classification.

Additionally, the usability of the documentation systems used, namely the standard clinical documentation systems and the AI‐based digital system, will be assessed in both the control and intervention groups. Usability will be evaluated using the two‐item UMUX Lite questionnaire with a 7‐point Likert scale (Lewis et al. 2013) because of its brevity (Borsci et al. 2020) and comparability with other studies and the SUS scale (Lewis et al. 2013).

### 2.11 Data Collection and Management

The pre‐intervention data collection period began in May 2023 and continued until the required sample size was reached in January 2024. Following the completion of AI development in the technical sub‐study, we began training nurses and initiated the intervention phase with the corresponding observations and surveys in July 2024. Observations, on‐site wound assessments, and surveys of the nurses are conducted by trained nursing scientists using a digital questionnaire on tablets through the Lime Survey tool (Limesurvey GmbH 2024). The collected data is anonymized and stored on secure, access‐restricted servers available only to the research team.

To ensure that the documentation created can be linked to the observations while maintaining data confidentiality and security, a unique identifier is generated in the AI‐based application and recorded on the observation sheet. Neither this ID nor the data collected during the wound documentation, observation, and survey can be used to identify the patient or nurse. All collected data is stored in a password‐protected folder accessible only to the study team. Consent forms and confirmation of receipt of the voucher are stored securely and are also only accessible to the study team. Upon completion of data collection, the data will be exported and statistically analyzed using SPSS version 28.0.0.0 (IBM Corp 2021). Given the study's small sample size, relatively low complexity, and clearly defined endpoints, the establishment of a data monitoring committee is not deemed necessary.

### 2.12 Analysis Plan

Once data collection is complete, we will use multiple linear regression models to examine the effects of the AI‐based digital system on the duration of wound assessment, dressing change, documentation, perceived task load, and guideline adherence. We will calculate an individual linear regression model for each dependent variable and incorporate potential covariates such as the nurses’ professional experience, wound severity, and the general load of the shift. Nominal and ordinal variables (use of the AI demonstrator, severity of the wound, general burden of the shift, and guideline adherence) will be dummy coded. We will then assess each model for linearity, homoscedasticity, and the absence of multicollinearity to satisfy the assumptions of linear regression. If these conditions are met, we will proceed with standard multiple linear regression, incorporating all independent variables as predictors. The f‐test will be used to examine the extent to which the independent variables predict the dependent variable, with R² indicating how much of the variance in the dependent variable can be explained by the independent variables. The t‐test will assess the significance of the predictors. To determine the specific contribution of the use of the demonstrator, we will calculate the explanatory power for both the full regression model and a reduced model without the demonstrator use variable, using the difference in explanatory performance to isolate the effect attributable to the demonstrator use. Datasets with missing socio‐demographic data for nurses, used only to describe the sample and not part of the statistical analysis, will still be included in the analysis. However, datasets with missing values for independent variables will be excluded entirely, and datasets with missing values for dependent variables will be excluded only from the specific model where the data is missing. The significance level is set at 0.05. As this is an exploratory study and a separate model is calculated for each dependent variable, no correction for alpha error is required. To analyze the interrater reliability of the observers, we will use Kendall's rank correlation coefficient (Kendall's tau). To determine the performance of the AI‐based application in the clinical setting, we will calculate its accuracy in predicting wound type and wound classification, using the on‐site assessment of a study team member as ground truth. Additionally, we will descriptively evaluate the usability of both the standard clinical documentation systems and the AI‐based application in terms of central tendency, variability, and distribution.

All statistical analyzes will be performed using SPSS version 28.0.0.0 (IBM Corp 2021).

## 3 Discussion

This study protocol outlines a non‐randomized controlled trial designed to evaluate the effects of an AI‐based application on nursing wound assessment, care, and documentation, including its accuracy in predicting wound type and classification in a clinical setting. By integrating this application into nursing practices, we anticipate reductions in the duration of these processes, a decreased task load for nurses, and increased adherence to guidelines.

The technical feasibility of AI‐supported wound assessment and documentation has been established in recent studies (Ohura et al. 2019), as well as the technical evaluation (Howell et al. 2021) and its positive effects on the quality of wound documentation, wound healing, and patient compliance (Barakat‐Johnson et al. 2022). However, research into the application of AI in nursing has been limited, with few studies exploring its clinical potential beyond development (O'Connor et al. 2023). To our knowledge, the KIADEKU overall project is the first research and development project to explicitly incorporate nursing guidelines and specific nursing requirements in the development of an AI‐based application. In this interventional sub‐study, we adopt a user‐centered approach to evaluation, primarily exploring the AI's potential to support nurses in managing and documenting wound care while enhancing guideline adherence.

The selection of standardized and validated questionnaires such as the NASA TLX (Hart 2006) and UMUX lite (Borsci et al. 2020) to measure nurses’ task load and the usability of the documentation systems ensures high quality and comparability with other studies. Since no suitable instrument exists to measure guideline adherence in wound care observation, we identified specific criteria in an expert workshop to ensure both construct and content validity. These criteria informed our definition of standardized observation procedures and criteria to maintain objectivity.

Potential challenges in implementing the AI‐based application in a clinical setting include technical issues, such as ensuring full Wi‐Fi coverage, and meeting hygiene requirements, such as disinfecting tablets. Another common hurdle in technology intervention studies is user acceptance. However, in the realm of technologies that support nursing tasks, studies have shown high technology acceptance (Zöllick et al. 2020). We therefore anticipate high acceptance due to the simplification of wound care and documentation processes. To further enhance user acceptance, local wound experts and ward managers have been involved in the development of the application and will be key users during its implementation on the wards. Additionally, the implementation will be supported by comprehensive training measures to ensure safe use and handling of the application and AI predictions. This is crucial, especially when addressing incorrect or inaccurate predictions made by AI and the ethical challenges that arise in biomedical AI (Mittelstadt et al. 2016).

As this study is monocentric, focusing only on nurses at one university hospital, the external validity is limited. Another limitation is the constrained resources of the project, which preclude the use of a randomized study design and may introduce slight biases due to the consecutive and non‐randomized observation of control and intervention groups, as well as the absence of blinding. Moreover, the organization of information events and recruitment of nursing staff may heighten awareness of pressure injury and IAD care, potentially influencing care and documentation and thus the results of the study. Organizational delays, which are inevitable due to clinical processes, may also introduce bias. We also acknowledge that factors such as ward affiliation might influence outcomes, but were not included in this exploratory pilot study due to the complexity of the models required and the limited sample sizes. For this reason, only the most crucial factors, from an expert perspective, were included in the analysis.

This study is an exploratory pilot study. Further research is required to draw general conclusions about its potential to support nursing wound management, particularly regarding patient‐related outcomes and the impact on nurses’ critical thinking and workflows. Additionally, we aim to enhance understanding of AI and the valuable contributions nurses can make with their data, and to actively involve nurses in the development and implementation of AI to realize its potential in nursing.

As for the current progress of the study, data collection for the control group was completed in January 2024. Following the development of the AI and its integration into the prototype demonstrator, nurses from the selected wards were trained, and data collection for the intervention group commenced in July 2024. This phase of data collection is still ongoing and is expected to be completed by spring 2025. To date, no adjustments to the intervention study methodology have been necessary.

In conclusion, this interventional pilot study investigates the potential of AI in nursing wound assessment, management, and documentation. The findings could contribute to the further development of AI applications in nursing wound care and other nursing scenarios.

## Author Contributions

PH, RV, FU developed the theoretical concept and design of the study. BA and AM developed the AI and the AI application. HB, FU and AM applied for funding. PH wrote the manuscript with the support of RV. FU, HB, MK, BA, TM, AM and KJ critically revised the draft and contributed to the final writing of the paper. All authors read and approved the final manuscript.

## Conflicts of Interest

The authors declare no conflicts of interest.

## Data Availability

The data that support the findings of this study are available from the corresponding author upon reasonable request.

## Endnotes

1 German abbreviation for Artificial Intelligence.

2 German term for pressure injury.
